# A safe and effective protocol for postdilution hemofiltration with regional citrate anticoagulation

**DOI:** 10.1186/s12882-024-03659-y

**Published:** 2024-07-09

**Authors:** Thomas Dimski, Timo Brandenburger, Christian Vollmer, Detlef Kindgen-Milles

**Affiliations:** Department of Anesthesiology, University Hospital Duesseldorf, Heinrich-Heine University Duesseldorf, Moorenstr. 5, Duesseldorf, 40225 Germany

**Keywords:** Acute kidney injury, Continuous renal replacement therapy, Hemofiltration, Citrate anticoagulation, Intensive care medicine

## Abstract

**Background:**

Regional citrate anticoagulation (RCA) is recommended during continuous renal replacement therapy. Compared to systemic anticoagulation, RCA provides a longer filter lifespan with the risk of metabolic alkalosis and impaired calcium homeostasis. Surprisingly, most RCA protocols are designed for continuous veno-venous hemodialysis or hemodiafiltration. Effective protocols for continuous veno-venous hemofiltration (CVVH) are rare, although CVVH is a standard treatment for high-molecular-weight clearance. Therefore, we evaluated a new RCA protocol for postdilution CVVH.

**Methods:**

This is a monocentric prospective interventional study to evaluate a new RCA protocol for postdilution CVVH. We recruited surgical patients with stage III acute kidney injury who needed renal replacement therapy. We recorded dialysis and RCA data and hemodynamic and laboratory parameters during treatment sessions of 72 h. The primary endpoint was filter patency at 72 h. The major safety parameters were metabolic alkalosis and severe hypocalcemia at any time.

**Results:**

We included 38 patients who underwent 66 treatment sessions. The mean filter lifespan was 66 ± 12 h, and 44 of 66 (66%) filters were patent at 72 h. After censoring for non-CVVH-related cessation of treatment, 83% of all filters were patent at 72 h. The delivered dialysis dose was 28 ± 5 ml/kgBW/h. The serum levels of creatinine, urea and beta2-microglobulin decreased significantly from day 0 to day 3. Metabolic alkalosis occurred in one patient. An iCa^++^ below 1.0 mmol/L occurred in four patients. Citrate accumulation did not occur.

**Conclusions:**

We describe a safe, effective, and easy-to-use RCA protocol for postdilution CVVH. This protocol provides a long and sustained filter lifespan without serious adverse effects. The risk of metabolic alkalosis and hypocalcemia is low. Using this protocol, a recommended dialysis dose can be safely administered with effective clearance of low- and middle-molecular-weight molecules.

**Trial registration:**

The study was approved by the medical ethics committee of Heinrich-Heine University Duesseldorf (No. 2018-82KFogU). The trial was registered in the local study register of the university (No: 2018044660) on 07/04/2018 and was retrospectively registered at ClinicalTrials.gov (ClinicalTrials.gov Identifier: NCT03969966) on 31/05/2019.

## Background

Acute kidney injury (AKI) is a frequent complication in intensive care units (ICUs), and renal replacement therapy (RRT) is required for approximately 15–20% of all ICU patients [1]. In critical ill patients, continuous renal replacement therapy (CRRT) is frequently used because it provides good hemodynamic stability and volume control [2]. For effective anticoagulation of the extracorporeal circuit and to avoid bleeding complications, regional citrate anticoagulation (RCA) is recommended [3]. A variety of RCA protocols have been published; however, the majority of studies have investigated continuous veno-venous hemodialysis (CVVHD) [4] or hemodiafiltration (CVVHDF) [5, 6]. In contrast, effective protocols for continuous veno-venous hemofiltration (CVVH) [7, 8] are rare and filter lifespans during CVVH are often shorter than those during other treatment modes. This is surprising because hemofiltration is frequently used and may provide better middle molecule clearance than other CRRT techniques [9]. Therefore, we investigated in this study a citrate protocol for postdilution hemofiltration designed to achieve effective regional anticoagulation, a sustained filter lifespan and effective small- and middle-molecule clearance without a clinically relevant risk of adverse metabolic effects.

## Methods

This was a single-center prospective interventional study of a new RCA protocol for postdilution CVVH. The study was performed in the level 3 surgical ICU of an university hospital.

### Patients

The inclusion criteria for patients were treatment in the ICU and AKI stage III according to the KDIGO (Kidney Diseases Improving Global Outcomes Initiative) criteria from 2012 [10] and the need for renal replacement therapy according to local guidelines (i.e., hyperkalaemia, fluid overload resistant to diuretics, metabolic acidosis, or clinical signs of uremia). Patients were included after written informed consent. Consent was given by the patient themselves. In the case of incapacitated patients, consent was given by their legal representative.

The exclusion criteria were age < 18 y, pregnancy or breast feeding, severe lactic acidosis (i.e., lactate > 10 mmol/L and pH < 7.2 for longer than 6 h) or known intolerance to citrate.

### Intervention

CRRT was performed as postdilution CVVH using a commercially available device (PLASAUTO SIGMA™, Fa. Diamed Medizintechnik, Cologne, Germany; Software Version LCD 3.10Ar, CTRL 3.04Bn, MON 3.01c) and a synthetic biocompatible polysulfone hemofilter with a surface area of 1.8 m^2^ and a sieving coefficient for beta-2-microglobulin of 0.8 (APS-18H, Fa. Diamed Medizintechnik, Cologne, Germany).

A 4% trisodium citrate dihydrate solution (136 mmol/L, Fresenius Kabi AG, Bad Homburg, Germany) was infused into the arterial part of the hemofiltration circuit close to the connection of the blood circuit to the dialysis catheter. The target concentration for citrate was 3.5 mmol/L to yield an iCa^++^ concentration below 0.35 mmol/L. To lower iCa^++^ to these levels, for a given blood flow of 100 ml/min, a citrate flow of 2.6 ml/min (ratio of citrate flow/blood flow = 1:39) is needed. We calculated adjustments in citrate flow according to the level of postfilter iCa^++^.

A 10% calcium gluconate solution (Calciumglukonat 10%, BBraun, Melsungen, Germany) was infused into the venous part of the circuit close to the dialysis catheter to restore calcium loss via the ultrafiltration fluid. We estimated a loss of 50% of citrate calcium complexes via the hemofilter so that a calcium substitution of approximately 2 mmol per liter ultrafiltration was necessary to maintain a neutral calcium balance. This resulted in a calcium gluconate flow rate of 9 ml per liter of ultrafiltrate with corresponding adjustments according to the level of iCa^++^ as measured from arterial blood samples.

The substitution fluid was calcium-free and had a reduced concentration of bicarbonate to compensate for bicarbonate generation from citrate (Biphozyl™, Baxter-Gambro, Lund, Sweden; sodium 140 mmol/L, potassium 4.0 mmol/L, Mg 0.75 mmol/L, Cl 122 mmol/L, HPO_4_- 1 mmol/L, HCO_3_- 22 mmol/L). The initial ratio of blood flow (ml/min) to total net effluent (ml/h) was 1:20.

With these default settings, treatment started. During the treatment, clinicians were advised to follow the protocol recommendations. The citrate and calcium substitution flow rates were adjusted depending on the levels of postfilter iCa^++^ and iCa^++^ measured in the arterial blood samples. Blood and substitution fluid flow rates were adjusted according to the acid base status.

#### Adjusting citrate dose for control of postfilter iCa^++^

In the case of high postfilter iCa^++^ (> 0.35 mmol/l), the citrate/bloodflow ratio was increased (e.g., from 1:39 to 1:37).

In the case of low postfilter iCa^++^ (< 0.3 mmol/l), the citrate/bloodflow ratio was reduced (e.g., from 1:39 to 1:41).

#### Adjusting systemic iCa^++^

In the case of high systemic iCa^++^ (> 1.3 mmol/l), calcium substitution was reduced by 3 ml/h. Patients with low systemic iCa^++^ (< 1.0 mmol/l) were treated with a bolus of 3 ml of 10% calcium gluconate, followed by an increase in the calcium substitution rate of 3 ml/hr.

#### Adjusting the pH

In the case of metabolic acidosis (arterial pH < 7.35), a three-step approach was recommended:

1. The blood flow (and thus the citrate dose given to the patient) was increased by 10%.
2. If the increase in pH after 4 h was inadequate, blood flow increased by another 10%.
3. If acidosis is not controlled by steps 1 and 2, the substitution flow rate should be reduced by 10% (this way eliminating less bicarbonate).

In case of metabolic alkalosis (arterial pH > 7.45), a three-step approach was recommended:

1. Reduce blood flow by 10%
2. If pH decrease was inadequate after 4 h increase substitution flow by 10% (i.e. because of hemoconcentration blood flow should not be reduced by more than 10%)
3. If alkalosis is not controlled by steps 1 and 2, decrease the citrate flow rate by 10% independent of blood flow.

### Data acquisition and endpoints

We recorded patient demographic data, the reason for ICU admission, the major cause of AKI, hemodynamic data, the need for catecholamine therapy and mechanical ventilation, the SOFA score on the day of the first RRT treatment and a variety of laboratory data.

Blood samples were taken from the arterial line of the patient and analysed in the central laboratory (serum chemistry, blood count, coagulation parameters, total Ca^++^) once daily, or at the point of care (arterial blood gas analysis including pH and bicarbonate levels and postfilter iCa^++^; ABL 850^TM^, Radiometer, Copenhagen, Denmark) every 6 h.

We recorded data on the delivery of CVVH, including blood flow, total net effluent (the sum of net ultrafiltration and replacement fluid including the applied citrate), filter lifetime, and reasons for discontinuation of treatment. We calculated the delivered dialysis dose (ml/kg BW/h).

The primary endpoint was hemofilter patency at 72 h. The safety endpoints were metabolic alkalosis (pH > 7.5) and arterial iCa^++^  < 1.0 mmol/L.

Statistical analysis was performed using descriptive and inferential methods. To calculate the statistical significance of the metabolic effects, a t test for dependent samples was used. The Kaplan‒Meier method was used for the statistical analysis of the time to clot during the filter lifespan. Statistical analysis was performed with GraphPad Prism software version 7.03. All the data are presented as the mean ± standard deviation (mean ± SD). *P* < 0.05 was considered significant.

## Results

### Patients

**Fig. 1:**
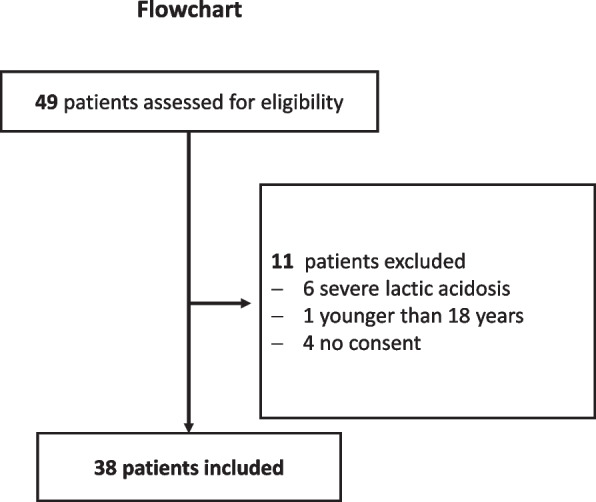
Trial flowchart

From January 2019 to August 2020, we studied 66 hemofiltration circuits in 38 patients (shown in Fig. 1). All patients provided written informed consent. Patients were admitted to the intensive care unit after major surgical interventions (cardiosurgical, vascular or general abdominal surgery). The demographic data and baseline characteristics are shown in Table 1. The etiologies of AKI were sepsis or septic shock (*n* = 19), cardiac failure/cardiogenic shock (*n* = 17), and hypovolemia caused by bleeding (*n* = 2). Overall hospital mortality was 63% (24/38).

**Table 1:** Demographic data of 38 patients (mean ± SD). The SOFA score was calculated at the start of renal replacement therapy

**Age [y]**	65 ± 10
BMI	29 ± 5
Height [cm]	172 ± 8
Weight [kg]	87 ± 16
SOFA Score	14 ± 4

### Filter lifespan and anticoagulation

**Fig. 2:**
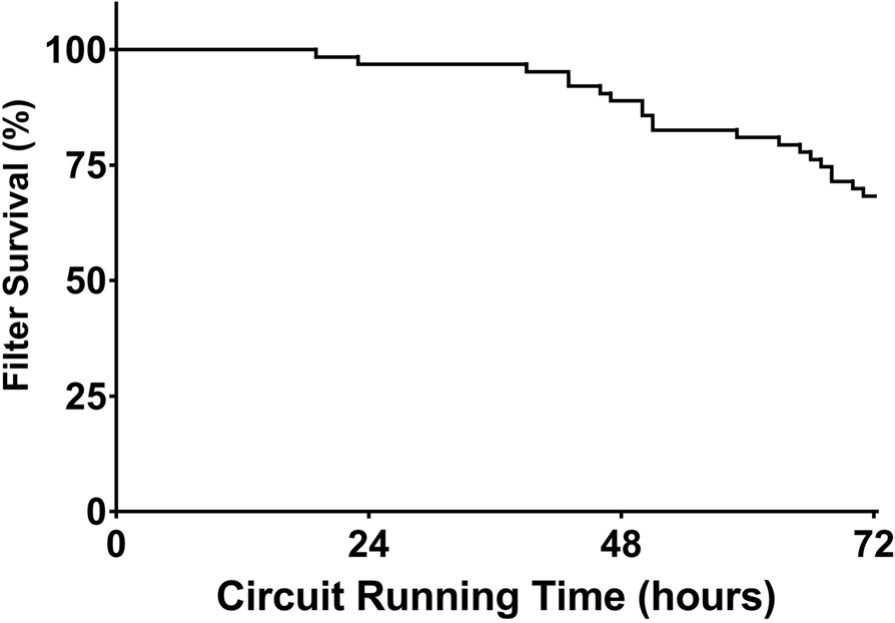
Kaplan–Meier survival curve for hemofilters. Data from 66 hemofiltration circuits

The mean filter lifespan was 66 ± 12 h. Early malfunction, i.e., a filter lifespan < 24 h, occurred in 3/66 circuits (4.5%). After 72 h, 44 of 66 (66%) filters were patent. The reasons for the termination of therapy were an attainment of the filter lifespan of 72 h in 44 of 66 (66%), clotting in 9 of 66 (14%), and reasons not related to CRRT in 13 of 66 (20%). After exclusion of these 13 cases, 44 of 53 (83%) of all the filters were patent after 72 h of circuit operation. Figure 2 shows the Kaplan‒Meier estimates of filter lifespan for all patients.

**Fig. 3:**
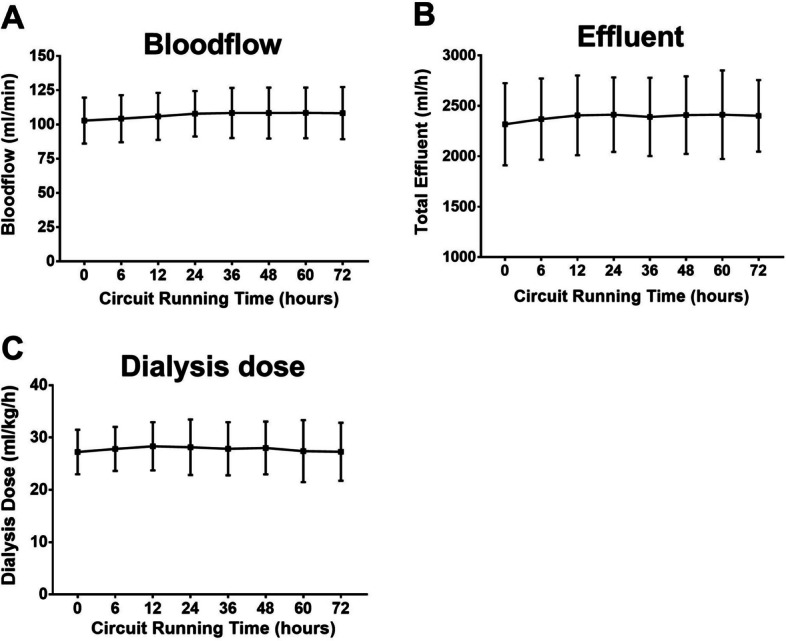
Parameters of citrate anticoagulation. Citrate flow rate, postfilter iCa^++^, calcium substitution rate and systemic iCa^++^. Data from 66 hemofiltration circuits. Mean ± SD

Figure 3 shows the parameters of citrate anticoagulation, i.e., the flow rates of 4% trisodium citrate for anticoagulation and 10% calcium gluconate for calcium substitution, and the effects of these applications on postfilter ionized calcium (iCa^++^) and systemic iCa^++^. During 72 h of treatment, there was a small but nonsignificant increase in the citrate flow rate to maintain the postfilter iCa +  + within the target range. The substitution rate of 10% calcium gluconate did not change over 72 h, and the mean systemic iCa^++^ levels were always within the normal range.

We administered intravenous unfractionated heparin to 35 of 38 patients for thrombosis prophylaxis only. The standard dose for thrombosis prophylaxis is 10,000 IU/d. Heparin-induced thrombocytopenia type II (HIT-II) was diagnosed in three of these patients. These patients were treated with argatroban, but due to the high risk of bleeding, this treatment did not effectively prolong the aPTT. The INR increased, and thrombocyte counts decreased before RRT initiation due to underlying disease. There was no significant difference between the start of RRT and 72 h (shown in Table 2).

**Table 2:** Thrombocyte counts and coagulation parameters on day 0 and day 3 of hemofiltration therapy. No significant difference occurred between day 0 and day 3. The data were obtained from 66 hemofiltration circuits (mean ± SD)

	**Day 0**	**Day 3**	***P***
Thrombocytes [cells/µl]	152,000 ± 114,000	162,000 ± 116,000	0.1375
aPTT [s]	38 ± 10.0	41 ± 10.1	0.1078
Prothrombin time [%]	64 ± 12.8	62 ± 12.8	0.3205
INR	1.32 ± 0.21	1.36 ± 0.21	0.6566

### Treatment efficacy

**Fig. 4:**
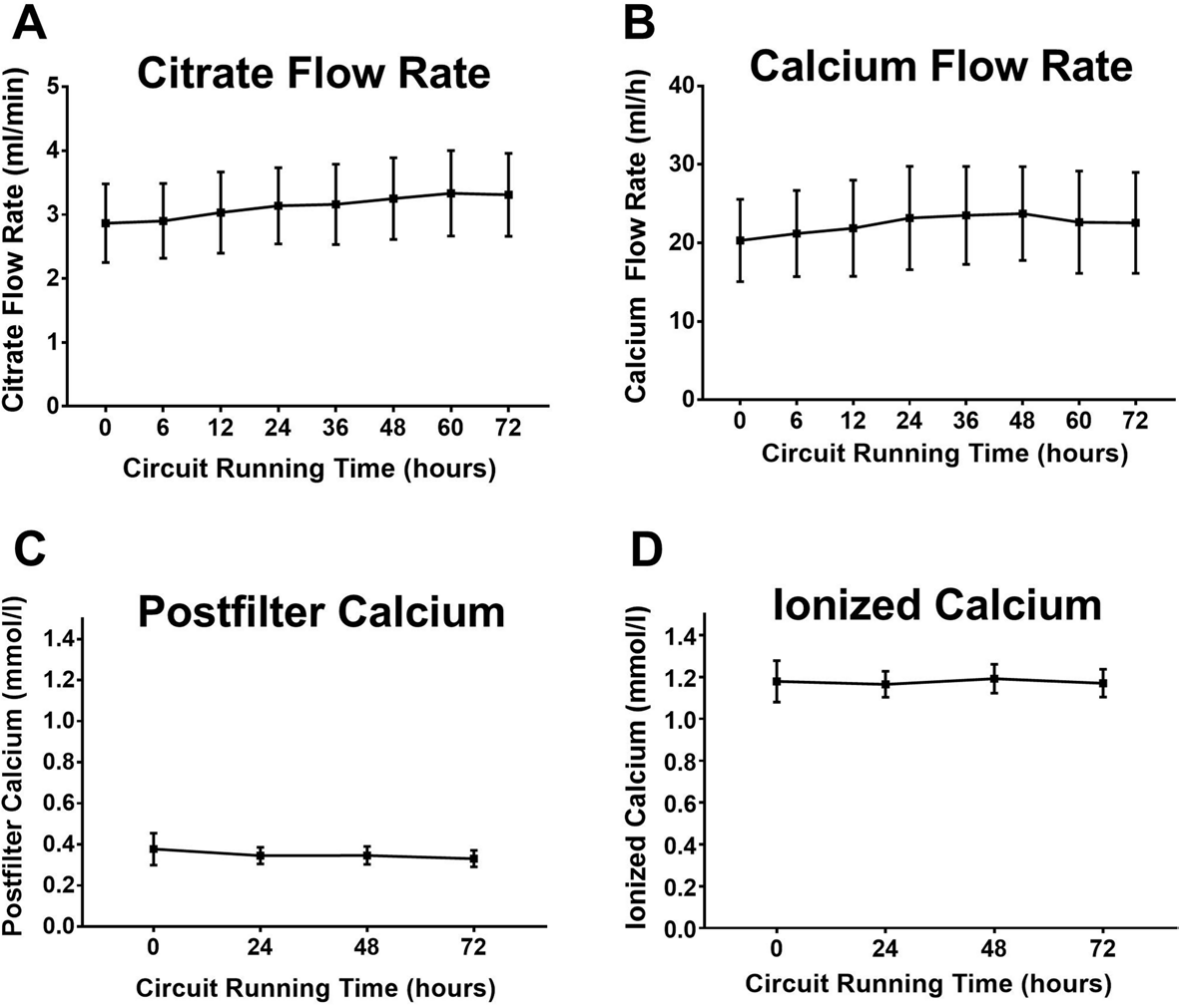
Blood flow [QB], total effluent [ml/min], and delivered dialysis dose [ml/kg BW/min]. Data from 66 hemofiltration circuits. Mean ± SD

Figure 4 shows the treatment efficacy, i.e., blood flow, total net effluent, and delivered dialysis dose. The mean blood flow was approximately 110 ml/min, and the total net effluent was approximately 2400 ml/min. The delivered dialysis dose was 28 $\pm$ 5 ml/kg/h, which is within the dose recommendations of current guidelines.

**Fig. 5:**
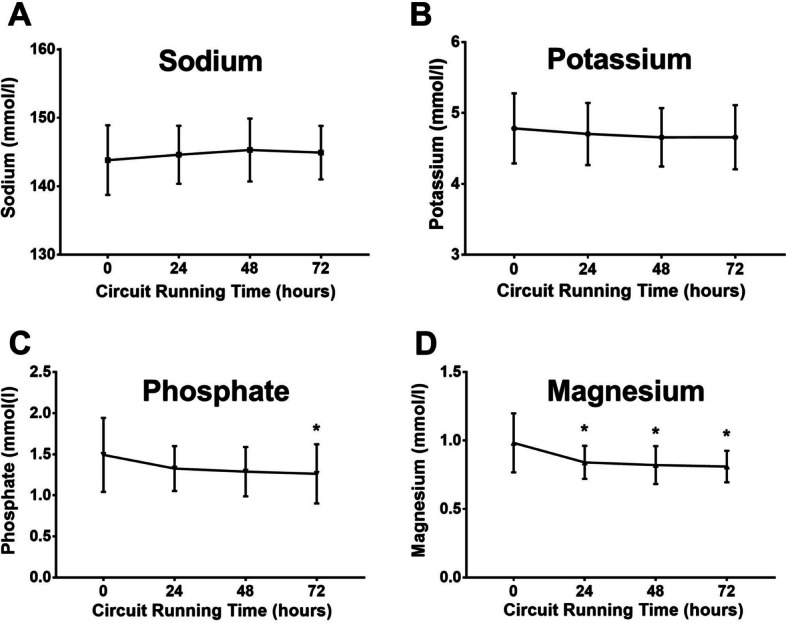
Acid‒base status during the 72 h treatment time. Data from 66 hemofiltration circuits. Mean ± SD

The mean pH and standard bicarbonate concentration were within the normal range after 72 h of treatment. A tendency towards metabolic alkalosis was not observed. The arterial CO_2_ levels were within the normal range and did not change during the 72 h treatment (Fig. 5).

The mean dialysis dose delivered was 28 ml/kg BW/h (Fig. 4). The serum concentrations of creatinine and urea decreased significantly from day 0 to day 3. Notably, the serum concentration of beta2-microglobulin, a typical middle molecule, also decreased significantly (Table 3).

**Table 3:** Serum levels of creatinine, urea, and beta2-microglobulin on day 0 and day 3 of hemofiltration therapy. All parameters decreased significantly from day 0 to day 3 of hemofiltration therapy. Data from 66 circuits for creatinine and urea and 33 circuits for beta-2-microglobulin due to missing data (mean ± SD)

	**Day 0**	**Day 3**	***p***
Creatinine [mg/dL]	1.92 ± 1.3	1.16 ± 0.6	< 0.0001
Urea [mg/dL]	83 ± 36	66 ± 27	0.0026
ß2-Microglobulin [mg/L]	11.0 ± 5.4	7.3 ± 3.3	0.0059

**Fig. 6:**
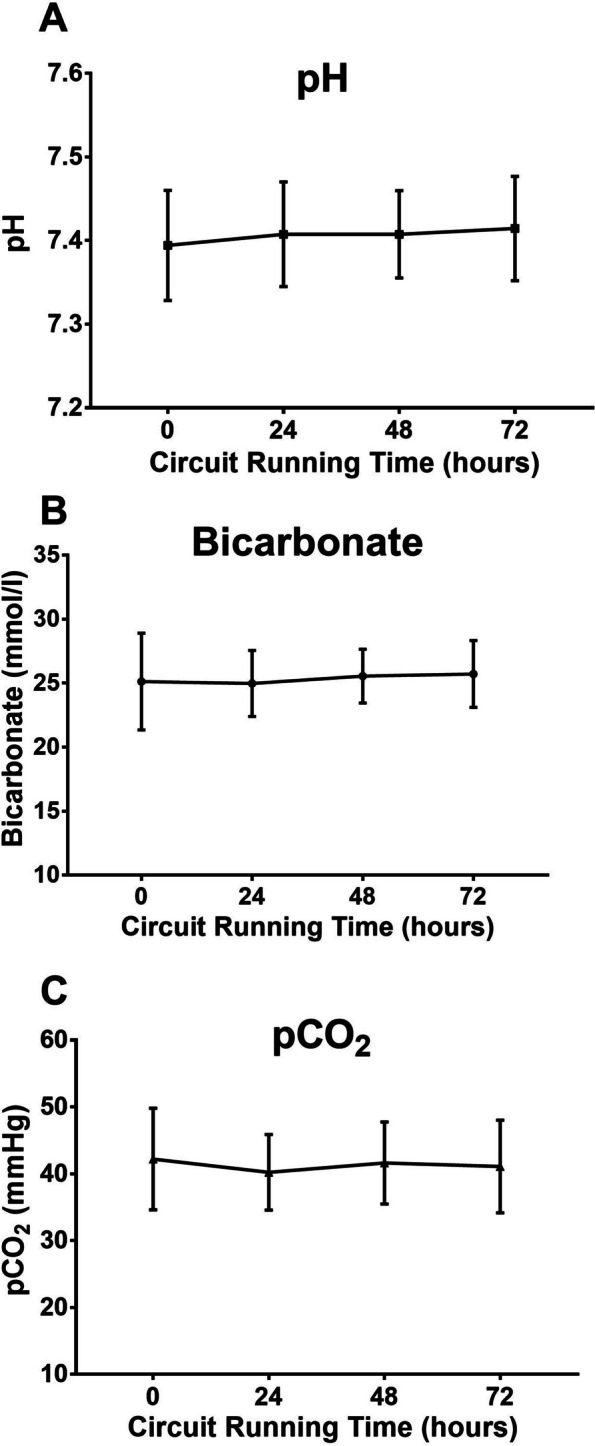
Serum levels of sodium, potassium, phosphate, and magnesium during 72 h of treatment. The mean sodium and potassium levels did not change and remained within the normal range over time. Serum levels of magnesium and phosphate decreased significantly from day 0 to 72 h of hemofiltration therapy. Data from 66 treatment episodes (mean ± SD)

The serum levels of sodium and potassium did not change over time. There was a significant decrease in magnesium and phosphate levels from day 0 to day 3, but the mean levels remained within the normal range (Fig. 6).

### Adverse effects

An iCa^++^ below 1.0 mmol/L was observed in 4 patients at one single time point. In these patients, the levels of iCa^++^ ranged between 0.97 and 0.89 mmol/L, and hypocalcemia always occurred within the first 12 h of CRRT. Normal iCa^++^ levels were restored immediately by bolus injection of calcium gluconate. Adverse effects were not observed.

Ionized hypercalcemia (> 1.35 mmol/l) was observed three times. Two patients had hypercalcaemia before the start of RRT. One patient developed ionized hypercalcemia (1.41 mmol/l) 48 h after the initiation of CVVH. After the reduction in the calcium substitution rate according to the protocol (a rate reduction of 3 ml/h), normal calcium levels were achieved within 6 h.

Elevated total calcium levels (> 2.42 mmol/l) were detected in 36 patients. Most of these reasons for elevated total calcium have remained elusive. Notably, we did not observe any signs of citrate accumulation, i.e., a ratio of total calcium/iCa^++^  > 2.5 was not detected. We observed 4 episodes of metabolic alkalosis (pH > 7.5 with elevated standard bicarbonate and normal PaCO2). Three patients were alkalotic before initiating CVVH. In these patients, the pH normalized within 16 h after initiation of CVVH with standard settings.

One patient developed metabolic alkalosis (pH 7.51) 48 h after starting CVVH under the default settings. After reducing the blood flow according to the protocol (reduction of blood flow by 10%), the pH normalized within 8 h.

## Discussion

We investigated the safety, efficacy, and feasibility of a protocol for regional citrate anticoagulation for postdilution hemofiltration in critically ill patients. The major observation is that this protocol provides sustained filter lifespans, with 66% of all circuits being patent at 72 h and a clotting-free filter survival of 83% at 72 h. An effective dialysis dose in line with current recommendations could be delivered. Accordingly, the serum levels of low- and middle molecules decreased significantly and pH control was excellent. Serious adverse effects were not observed.

Acute kidney injury is a serious complication in critically ill patients, and 15–20% of all ICU patients require renal replacement therapy (RRT) [1]. ICU-acquired AKI commonly occurs during severe disease, i.e., septic or cardiogenic shock or shock caused by hypovolemia. These patients often require vasopressor therapy; many patients are mechanically ventilated, and the metabolic burden is high. In such patients, CRRT is the therapy of choice because it provides better hemodynamic stability and volume control than do intermittent techniques [11].

A prerequisite for sustained filter lifespans and real continuous therapy is effective anticoagulation of the extracorporeal circuit. In this context, prospective randomized multicenter studies and aggregated data from meta-analyses have shown that the filter lifespan is significantly longer with RCA than with systemic anticoagulation [3, 12–14]. RCA can be applied for different CRRT techniques, i.e., CVVH, CVVHD, or CVVHDF. None of these techniques proved to be superior to others with regard to mortality. However, one advantage of postdilution CVVH is its high middle molecule clearance. Therefore, CVVH is preferred by many physicians [9]. Unfortunately, effective RCA protocols for postdilution CVVH are rare, and some such protocols are associated with clinically relevant adverse effects [15].

We studied this new protocol in a group of surgical ICU patients in whom AKI occurred after extended cardiac, vascular, or abdominal surgery. Therefore, effective systemic anticoagulation was not feasible. Patients were severely ill, as shown by a SOFA score of 14 at the start of RRT and a mortality rate of 63%. Thus, these patients are at high risk for adverse effects from any extracorporeal therapy.

We observed a mean filter lifespan of 66 h, two-thirds of circuits were still running at 72 h, and clotting occurred in 14% of all circuits only. These filter lifespans are longer than those of other protocols for CVVH or even CVVHDF. Kirwan et al. [16] published two trials on postdilution CVVH showing a median filter lifetime of < 27 h in their first study. In a second study [17], they analysed the optimal position of the catheter tip. Even with the tip position in the right atrium or the superior vena cava, the median filter life span was < 36 h. Morabito et al. [18] compared heparin anticoagulation and no anticoagulation using predilution CVVHDF to RCA in postdilution CVVH in 33 patients after cardiac surgery. They used an infusion of normotonic citrate (12 mmol/l in predilution) while the substitution fluid was infused in postdilution tchnique. This early study showed superiority of RCA compared to heparin and no anticoagulation. Filter life was significantly higher with RCA (49.8 ± 35.4 h) compared to heparin (30.6 ± 24.3 h) or no anticoagulation (25.7 ± 21.2 h). Of note, CVVHDF but not CVVH was used in predilution technique. In general, it is assumed that a predilution component prolongs filter life time due to lower haemoconcentration in the circuit. But the expected positive effect has not been proven. In Morabitos study the filter life under RCA was significantly longer compared to heparin. But compared to the filter life of 66 h in the present study the filter life of 50 h of RCA-CVVH in the Morabito study was 16 h shorter. This observation might be explained by a higher citrate target concentration of 3.5. mmol/L in our study compared to 3.0 mmol/L in the Morabito study. Stucker et al. [19] studied hemodiafiltration with two-thirds of the replacement fluids administered in predilution mode. Although hemoconcentration must have been low with more substitution fluid administered in predilution, the median filter lifespan was only 49 h. We administered a mean blood flow of 110 mL/min and a substitution flow of 2400 mL/min. Assuming a hematocrit of 30% the filtration fraction is close to 50%. In general, it is recommended that filtration fraction should be kept below 30% to avoid clotting [20]. However, the role of filtration fraction has been discussed recently [21], and the so-called end-of-filter haematocrit is likely more relevant than the filtration fraction itself. The authors conclude that the reliance on a single arbitrary parameter such as the filtration fraction lacks accuracy and validity. Most importantly, other factors, such as circuit design and the effects of (regional) anticoagulation, are much more important than the filtration fraction. Our observations are in line with these considerations since we observed an excellent filter lifespan despite a higher filtration fraction. In fact, our filter lifespan is much longer than that of many other protocols for CVVH [7, 16, 17] or CVVHDF [19].

Long filter lifespans were achieved with moderate – nonsignificant – adjustments of the citrate dose during the 72 h treatment time. In addition, relevant adjustments of calcium supplementation were not necessary. In settings with reduced availability of highly skilled staff or increased workload, a safe and easy-to-handle protocol requiring only a few adjustments may increase patient safety.

Current guidelines recommend prescribing a dialysis dose of 30 ml/kg BW/h to provide a delivered dose of 20–25 ml/kg BW/h [10]. We delivered a dose of approximately 28 ml/kg BW/h during the treatment, which effectively decreased the serum levels of small solutes such as creatinine and urea.

A potential benefit of convective blood purification is greater middle molecule clearance. For this reason, CVVH is still advocated by some physicians. Of note, recent developments in filter technology have led to the so-called medium cut-off or high cut-off filters [22, 23]. With these membranes, a variety of middle molecules (beta2-microglobulin, myoglobin, IL-6 and even to some amount free light chains) can be eliminated effectively using a CVVHD mode with RCA [24, 25].This way, the limitation of CVVHD in this regard can be overcome. Despite these improvements in technology no mortality benefit of convective over diffusive blood purification has been shown in AKI [26]. In contrast, in patients with ESRD, smaller studies showed an improved outcome with greater middle molecule clearance [27, 28], and an inverse relationship occurred between the amount of convective volume turnover and mortality [29]. Recently, a prospective randomized trial in 1360 ESRD patients showed a decrease in mortality from 21.9% to 17.3% (HR 0.77; 95% CI 0.65–0.95) in patients who received high-dose hemodiafiltration compared to those who received high-flux hemodialysis [30]. Certainly, these data cannot be transferred to AKI patients in general, but the potential benefits of greater middle molecule clearance in critically ill patients with a high burden of uremic and other toxins are worth discussing. Our treatment approach significantly reduced the serum levels of beta2-microglobulin and thus provided good middle-molecule clearance.

The delivered dialysis dose also stabilized the pH and serum bicarbonate, and acidosis was well controlled. In RCA, the infused citrate has two important functions: anticoagulation and buffering. In most protocols, approximately 50% of citrate is removed via the dialyzer, while 50% enters the systemic circulation [4]. Citrate is metabolized mainly in liver cells within the Krebs cycle. One mole of citrate yields 3 mol of bicarbonate. Thus, changing the citrate load during RCA may alter the pH [4]. The buffer load applied via citrate must be taken into account when choosing the substitution fluid. Therefore, our substitution fluid contained a bicarbonate concentration of 22 mmol/L, which is lower than that of conventional solutions, which often contain 35 mmol/L bicarbonate. In this way, the buffer load applied via citrate and the reduced bicarbonate concentration of the substitution fluid matched well, and the pH was controlled without the need for frequent adjustments.

We cannot extend these conclusions to patients with severe liver failure. In those, impaired citrate metabolism and altered citrate pharmacokinetics [31] may alter the metabolic situation in a way that acidosis is not controlled, and severe hypocalcemia may occur. Although RCA may be applicable in the majority of patients with mild to moderate liver failure – sometimes with adjusted settings [32, 33] – in those with very severe liver failure other techniques can be used. This includes intermittent therapies or even coupled plasmafiltration and adsorption – the latter way adding a liver detoxification component [34].

CVVH therapy affected the serum levels of various ions and phosphate. Citrate was delivered as a 4% trisodium citrate solution. Therefore, hypernatremia is a typical adverse effect of this treatment. Our substitution fluid contained a reduced sodium concentration, and thus, hypernatremia did not occur.

Potassium control is relevant in patients with AKI. We administered a substitution fluid containing 4 mmol/L potassium, and the serum potassium concentration was well controlled in all patients.

However, we noted a significant decrease in the serum levels of magnesium and phosphate from day 0 to day 3. Both hypomagnesemia and hypophosphatemia carry risks for patients, including cardiac arrhythmias [35, 36], increased mortality [35, 37], weaning failure and an increased rate of tracheostomies in mechanically ventilated patients [38]. The substitution fluid contained 0.75 mmol/L magnesium and 1 mmol/L phosphate. The magnesium levels were still close to the lower limit of the normal range (0.8 mmol/L) on day 3, and phosphate levels decreased but remained within the normal range (0.84–1.45 mmol/L). With treatment durations exceeding 72 h or dialysis doses higher than 28 ml/kg BW/h, a relevant decrease in these serum levels under the normal range might occur. Daily measurements of Mg and phosphate are therefore recommended during ongoing CRRT.

### Adverse effects

The adverse effects directly related to RCA are hypo- and hypercalcemia, metabolic alkalosis, and citrate accumulation.

We observed four episodes of an iCa^++^ below 1.0 mmol/L. The lowest serum level was 0.89 mmol/L, which is considered noncritical and is usually not related to relevant clinical effects. Hypocalcemia occurred early during the first 12 h of CVVH and was reversed immediately with i.v. calcium application. Decreased levels of iCa^++^ were not observed after 12 h.

We observed only one episode of hypercalcemia related to hemofiltration, which was reversed within a few hours after adjusting the calcium substitution rate according to the protocol.

In critically ill patients, with liver failure or lactic acidosis the citrate accumulation may occur. Citrate per se is not toxic, but failure to metabolize citrate may lead to decreased serum levels of iCa^++^, an increased demand for calcium substitution, and metabolic acidosis [39, 40]. We did not observe citrate accumulation, although the severity of disease in our population was high, as shown by a mean SOFA score of 14.

Another adverse effect of RCA is metabolic alkalosis, which occurs with an incidence between 3 and 29% in different protocols [8, 41–43]. We observed only one episode (1.5%) of metabolic alkalosis related to CRRT, and reversal was achieved within 16 h. Thus, the protocol is safe with regard to this complication.

### Strengths and limitations

The strength of this study is its prospective design, which allowed early detection and control of adverse effects. The protocol was easy to use and provides clear recommendations on how to react in the case of deviations from the target range of any parameter. Another strength is the population. All patients were severely ill, and thus had a high risk for adverse effects.

In all patients, effective systemic anticoagulation was not feasible. In patients without bleeding risk, adding a systemic anticoagulation either by non-fractionated or low molecular weight heparin may further prolong filter life.

Limitations of the study include its monocentric design and the limited number of treatments. However, for a first proof of concept, the size of the study population is sufficient. The setting was a surgical ICU, so patients from internal medicine or neurology were not recruited. Notably, the majority of patients underwent cardiac surgery and had a high number of comorbidities. We did not evaluate very high dialysis doses, which might be necessary in cases of severe hyperkalaemia or intoxication with dialyzable drugs (i.e., metformin, lithium, alcohols). However, for the treatment of severe hyperkalaemia or intoxication, treatment modalities such as intermittent hemodialysis are recommended.

We accepted a high filtration fraction for the sake of low blood flows. We did not measure total protein in every patient at every time point. Our calculation of the filtration is mainly based on the assumption of a hematocrit of approx. 30% which in fact is typical for the majority of ICU patients. Admittedly, therefore, our calculation of filtration fraction is less accurate. Finally, physicians and nurses were familiar with different RRT devices, and studies on AKI are part of their daily work. Thus, prior to introducing this protocol into less experienced settings, thorough teaching is mandatory.

## Conclusion

We describe a safe, effective and easy-to-handle protocol for RCA in postdilution CVVH. This RCA protocol provides a long filter lifespan and the delivery of recommended dialysis doses. In addition, there was a significant decrease in the levels of middle molecules, as shown by the decrease in the level of beta2-microglobulin, which is a surrogate marker of middle molecule clearance. We observed excellent control of the pH, only one case of metabolic alkalosis, and a small number of other adverse effects, none of which were serious.

In conclusion, this protocol can be recommended for regional citrate anticoagulation of postdilution hemofiltration in critically ill patients.

## Data Availability

The data that support the findings of this study are available from D. Kindgen-Milles (kindgen-milles@med.uni-duesseldorf.de) but restrictions apply to the availability of these data, which were used under license for the current study, and so are not publicly available. Data are however available from the author D. Kindgen-Milles upon reasonable request and with permission of the local Ethics Committee Duesseldorf.

## Abbreviations

AKI: Acute kidney injury
CRRT: Continuous renal replacement therapy
CVVH: Continuous veno-venous hemofiltration
CVVHD: Continuous veno-venous hemodialysis
CVVHDF: Continuous veno-venous hemodiafiltration
ICU: Intensive care unit
KDIGO: Kidney Diseases Improving Global Outcomes Initiative
RCA: Regional citrate anticoagulation
RRT: Renal replacement therapy
